# Electronic patient-reported outcome assessment at inpatient admission: a descriptive analysis of real-world data for 1423 radiotherapy patients

**DOI:** 10.1186/s13014-026-02947-1

**Published:** 2026-10-23

**Authors:** Maria Schollmeyer, Heike Schmidt, Jörg Andreas Müller, Dirk Vordermark, Thomas Nordhausen

**Affiliations:** 1 Institute of Health, Midwifery and Nursing Science, Medical Faculty, Martin Luther University Halle‑Wittenberg, Halle, Germany; 2 Department of Radiation Oncology, University Hospital Halle, Halle, Germany

**Keywords:** Patient-reported outcomes, Health-related quality of life, EORTC QLQ-C30, Electronic assessment, Implementation, Radiation oncology, Hospital admission

## Abstract

**Background:**

Patient-reported outcomes (PRO) capture patients’ self-reported and treatment-related experiences, including functional impairment, symptoms, and health-related quality of life (HRQOL). Although PRO have shown benefits in many studies and are recommended by guidelines, systematic PRO assessment is still rarely integrated into routine cancer care. This paper describes results of the long-term implementation of electronic PRO assessment (ePRO) in the routine workflow of a radiation oncology inpatient clinic by analyzing patients’ HRQOL at admission.

**Methods:**

Admission ePRO included the EORTC QLQ-C30. PRO data were analyzed descriptively and stratified by sex, age, and primary tumor diagnosis.

**Results:**

The sample included 1,423 patients. More than half reported clinically important burden in physical and emotional functioning and fatigue. Male patients tended to report better functioning and lower symptom burden. Older patients reported greater burden in physical functioning, appetite loss, and constipation but less burden in social functioning and financial problems. Global health status showed little to no differences by sex, age, or tumor diagnosis.

**Conclusions:**

The analyses showed substantial self-reported functional impairment and symptom burden at admission in inpatient radiation oncology and highlight the value of ePRO for identifying needs early, when patients’ clinical status is not yet well known to the care team. At the organizational level, these results can help set priorities for supportive care during hospitalization and support more efficient resource allocation.

**Trial registration:**

Not applicable, the analysis used only PRO data collected as part of routine care in the inpatient radiation oncology clinic (see also the declaration of no objection).

**Supplementary Information:**

The online version contains supplementary material available at https://doi.org/10.1186/s13014-026-02947-1.

## Background

Several national and international organizations and guidelines strongly recommend integrating patient-reported outcomes (PRO) into routine oncology practice [1–3]. PRO are patients’ direct reports of functioning, symptoms, and health-related quality of life (HRQOL), without healthcare professionals (HCP) interpretation [4]. They are usually collected via standardized questionnaires (Patient-Reported Outcome Measures, PROM) and provide valuable insights on how patients personally experience their cancer disease and therapy [5].

PRO assessment can improve identification of cancer- and treatment-related burden because patients often perceive symptoms earlier and as more severe than HCP do, who may underestimate less visible symptoms such as fatigue [5, 6]. In this context, PRO simplify and standardize the monitoring and documentation of relevant issues, such as symptoms and functional impairments, and graded response options make changes in individual health status more visible and the information more precise [5, 7, 8]. Electronic PRO assessment (ePRO) using digital devices further increases the usability of PRO data in routine practice by automating processes such as real-time score calculation and graphical displays that highlight results requiring attention, e.g., via color coding [9]. Several studies indicate that PRO assessment benefits cancer treatment and care, including improved symptom management, patient satisfaction, and HRQOL [10–12]. Even “hard” outcomes such as unplanned hospital admissions and overall survival may improve when PRO data are used to guide clinical care [10, 13].

Despite these benefits, systematic PRO assessment remains largely confined to clinical research and is not routinely implemented in practice [7, 14, 15]. Consequently, most PRO data come from clinical trials and may not fully reflect real-world practice (e.g., additional staff and study-specific procedures) [16]. Real-world implementations can generate large samples and show whether PRO assessment workflows and findings are reproducible under routine conditions.

Accordingly, this paper describes the results of long-term ePRO implementation in a radiation oncology inpatient clinic by analyzing patients’ HRQOL at admission.

## Methods

### Project design, setting and sample

We conducted a descriptive analysis of ePRO data collected as part of routine care in the radiation oncology inpatient clinic (34 beds) at the Krukenberg Cancer Center, University Hospital Halle (Saale), Germany. Stepwise ePRO implementation occurred between February 2019 and February 2021; details of the implementation process have been published previously [17]. Assessment is offered to all admitted patients cognitively able to complete the questionnaires. The project was approved by the Ethics Committee of the Medical Faculty, Martin Luther University Halle-Wittenberg, via a declaration of no objection (January 8, 2019; retrospective process no. 2022-061).

### Instrument and procedure of ePRO

The EORTC QLQ-C30 [18], provided by the EORTC Quality of Life Group, is among the most widely used PROM [7, 19] and utilized to assess HRQOL at the time of hospital admission. It includes 28 items answered on a 4-point Likert scale (not at all, a little, quite a bit, very much) and 2 items answered on a 7-point scale, grouped into 15 functioning and symptom scales, each scored from 0 to 100 using the algorithms described in the EORTC QLQ-C30 Scoring Manual [20]. Higher functional-scale scores indicate better functioning, whereas lower symptom-scale scores indicate less symptom burden.

PRO data are collected electronically via touchscreen using either a mobile tablet or a fixed bedside monitor (patient entertainment system), both usable independently by patients. Dedicated student assistants introduce patients to the ePRO and provide support as needed.

Data collection and interpretation are performed with the CHES (Computer-based Health Evaluation System) software, integrated into the clinical information system (KIS). Results are displayed as bar charts in real time and are accessible directly in the KIS via an IT interface without additional password entry. They are also available as an automatically updated PDF in the KIS as a new report to increase physician visibility. Staff can review results cross-sectionally (single time point) or longitudinally (across multiple assessments).

### Data analysis

Within this project, ePRO data were exported from CHES and analyzed descriptively using SPSS version 25. We included all cases from February 25, 2019, through June 14, 2024, if the completed items allowed calculation of at least one EORTC QLQ-C30 scale.

We applied the clinical importance thresholds defined by Giesinger et al. [19] to classify the number and percentage of patients by reported burden on 14 of the 15 QLQ-C30 scales (no/low burden; clinically important burden), excluding the global health status scale, for which no thresholds were reported. This scale is presented without color coding in the results. Furthermore, we calculated means for each scale.

In addition to the full-sample analysis, we conducted subgroup analyses by sex and age as well as primary tumor diagnosis. Age groups and group labels were defined based on the approach used by Andersen et al. to analyze functional differences between younger and older adults with cancer using the EORTC QLQ-C30 [21]. Given the heterogeneity of health conditions associated with increasing age, such as physical and mental decline and frailty, the subgroup of older adults aged ≥ 65 years, as defined by Andersen, was further divided into three categories [22]. This resulted in five analyzed age subgroups: young adults (18–39 years), middle-aged adults (40–64 years), young-old (65–74 years), middle-old (75–84 years), and very old (≥ 85 years). For stratification by primary tumor diagnosis, all malignancy groups with more than 50 patients were included.

## Results

**Fig. 1:**
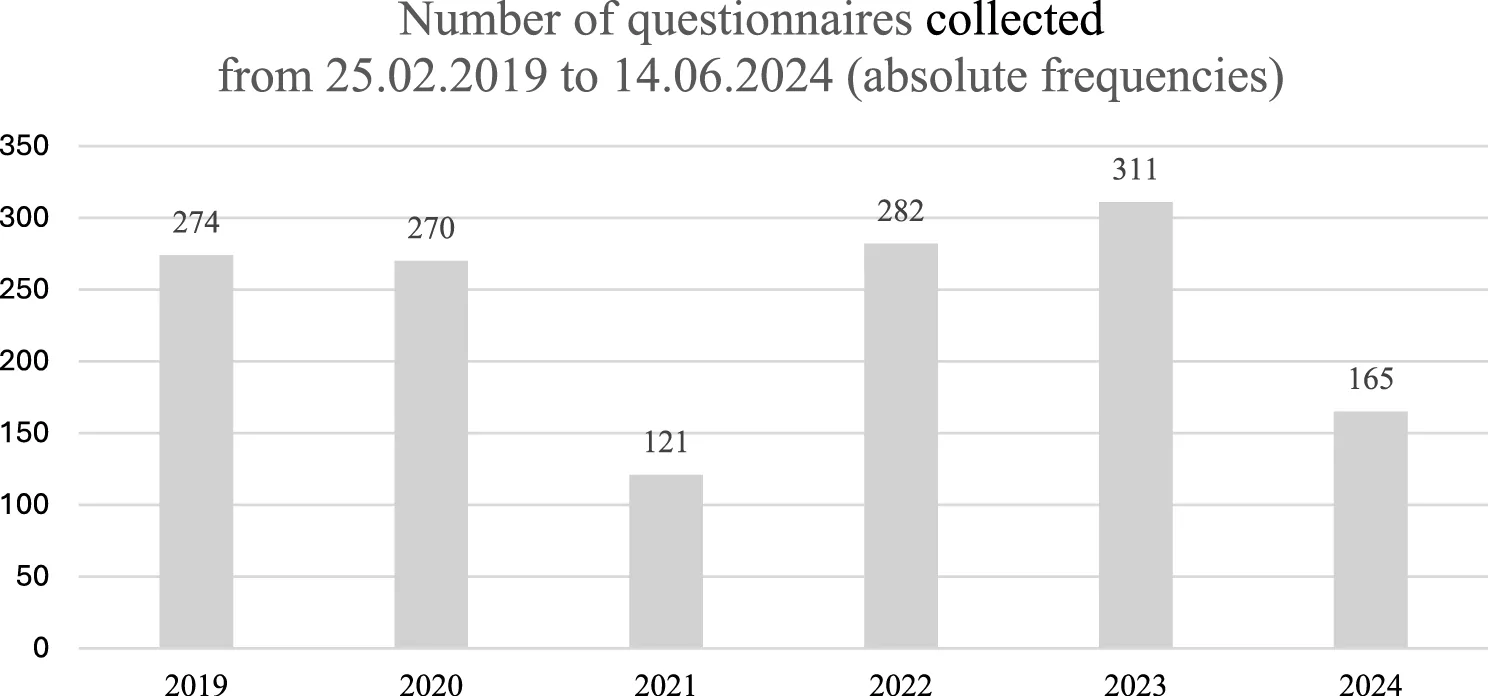
Timeline of EORTC QLQ-C30 data collection at inpatient radiation oncology admission (*n* = 1,423 patients)

Between February 25, 2019, and June 14, 2024, 5,882 patients were admitted to the radiation oncology clinic. Of these, 1,423 were included in the analysis, representing 24.2% of all admissions (Fig. 1).

### Patient characteristics

Approximately two-thirds of participants were male and one-third were female with a mean age of 66.0 ± 11.5 years at the time of ePRO completion. The most frequent age group was 40–64 years (> 40%), followed by 65–74 and 75–84 years. Patients younger than 40 and older than 84 years each made up < 6% of the sample. Head/neck and lung cancer were the most common diagnoses, together accounting for more than half of all cases. Table 1 summarizes patient characteristics.

**Table 1:** Characteristics of the patients (n = 1,423) included in the analysis of EORTC QLQ-C30 data at inpatient radiation oncology admission

Variable						
**Sex, n**(%)	**Total (n=1423)**					
Male	909	-63.9				
Female	514	-36.1				
Missing	0	0				
	**Total (n=1423)**		**Male (n=909)**		**Female (n=514)**	
Age*, years, Mean±SD (min-max)	66.0 ± 11.5 (23-97)		66.0 ± 10.6 (32-97)		66.0 ± 13.1 (23-94)	
**Age groups, years, n**(%)	**Total (n=1423)**		**Male (n=909)**		**Female (n=514)**	
Young adults (18-39)	21	-1.5	4	-0.4	17	-3.3
Middle-aged adults (40-64)	615	-43.2	415	-45.7	200	-38.9
Young-old (65-74)	427	-30	282	-31	145	-28.2
Middle-old (75-84)	299	-21	177	-19.5	122	-23.7
Very old (≥ 85)	61	-4.3	31	-3.4	30	-5.8
Missing	0	0	0	0	0	0
**Primary tumor, n**(%)	**Total (n=1423)**		**Male (n=909)**		**Female (n=514)**	
Head/neck	422	-29.7	318	-35	104	-20.2
Lung	398	-28	269	-29.6	129	-25.1
Colon/rectum	127	-8.9	85	-9.4	42	-8.2
Upper gastrointestinal (GI) tract	86	-6	69	-7.9	17	-3.3
Female genital organs	77	-5.4	0	0	77	-15
Skin	49	-3.4	27	-3	22	-4.3
Brain	43	-3	23	-2.5	20	-3.9
Breast	43	-3	0	0	43	-8.4
Kidney/urinary organs	39	-2.7	27	-3	12	-2.3
Lymphatic and hematopoietic tissue	27	-1.9	14	-1.5	13	-2.5
Prostate/other male genital organs	25	-1.8	25	-2.8	0	0
Liver/bile duct system	16	-1.1	11	-1.2	5	-1
Thyroid/other endocrine glands	16	-1.1	8	-0.9	8	-1.6
Mesothelial/soft tissue	10	-0.7	7	-0.8	3	-0.6
Pancreas	9	-0.6	5	-0.6	4	-0.8
Bone/cartilage	2	-0.1	1	-0.1	1	-0.2
Unknown localization	21	-1.5	14	-1.5	7	-1.4
Other tumors	6	-0.4	1	-0.1	5	-1
Missing	7	-0.5	5	-0.6	2	-0.4

*age based on the range between date of birth and date of assessment

### Full sample

More than half of all included patients reported clinically important burden in physical functioning (*n* = 1,033; 72.6%), emotional functioning (*n* = 759; 53.3%), and fatigue (*n* = 747; 52.5%) (Table 2).

### Subgroup analysis by sex

Both groups had a mean age of 66.0 years (± 10.6 for men/13.1 for women). More than half reported clinically important burden in physical functioning (men and women), role functioning (women), emotional functioning (women) and in fatigue (women). Overall, a smaller percentage of men reported a clinically important burden on most scales, and the mean scores in this group were generally lower. The largest differences were observed in emotional functioning (14.3% fewer men rated burden as clinically important; mean scores 8.0 points higher among men), fatigue (14.1% fewer men rated burden as clinically important; mean scores 8.9 points lower among men), and insomnia (11.2% fewer men rated burden as clinically important; mean scores 8.1 points lower among men).

Among all scales, global health status showed the smallest difference in mean scores between sexes, by 0.6 points. Table 2 summarizes the analysis stratified by sex.

**Table 2:** Sex-stratified distribution according to Giesinger’s thresholds [19] and mean EORTC QLQ-C30 scale scores at inpatient radiation oncology admission (*n* = 1,423)

Functioning scales (higher scores are better)		Full sample *n* = 1423	Male *n* = 909	Female *n* = 514
**Physical functioning** n (%)	No/low burden	388 (27.3)	267 (29.4)	121 (23.5)
	Important burden	1033 (72.6)	640 (70.4)	393 (76.5)
	Missing	2 (0.1)	2 (0.2)	0 (0.0)
M ± SD		60.7 ± 29.1	62.5 ± 28.8	57.4 ± 29.2
**Role functioning** n (%)	No/low burden	732 (51.4)	480 (52.8)	252 (49.0)
	Important burden	679 (47.7)	421 (46.3)	258 (50.2)
	Missing	12 (0.8)	8 (0.9)	4 (0.8)
M ± SD		56.7 ± 36.1	58.1 ± 36.3	54.2 ± 35.6
**Emotional functioning** n (%)	No/low burden	646 (45.4)	459 (50.5)	187 (36.4)
	Important burden	759 (53.3)	438 (48.2)	321 (62.5)
	Missing	18 (1.3)	12 (1.3)	6 (1.2)
M ± SD		65.4 ± 25.4	68.3 ± 24.1	60.3 ± 26.9
**Cognitive functioning** n (%)	No/low burden	930 (65.4)	617 (67.9)	313 (60.9)
	Important burden	478 (33.6)	282 (31.0)	196 (38.1)
	Missing	15 (1.1)	10 (1.1)	5 (1.0)
M ± SD		79.7 ± 25.5	81.3 ± 24.0	76.8 ± 27.6
**Social functioning** n (%)	No/low burden	894 (62.8)	579 (63.7)	315 (61.3)
	Important burden	492 (34.6)	307 (33.8)	185 (36.0)
	Missing	37 (2.6)	23 (2.5)	14 (2.7)
M ± SD		65.6 ± 33.6	66.1 ± 32.9	64.7 ± 34.7
**Global health status** M ± SD		49.6 ± 22.0	49.4 ± 21.2	50.0 ± 23.4
**Symptom scales** (lower scores are better)				
**Fatigue** n (%)	No/low burden	667 (49.6)	472 (51.9)	195 (37.9)
	Important burden	747 (52.5)	431 (47.4)	316 (61.5)
	Missing	9 (0.6)	6 (0.7)	3 (0.6)
M ± SD		44.6 ± 29.9	41.4 ± 29.6	50.3 ± 29.6
**Nausea and vomiting** n (%)	No/low burden	991 (69.6)	667 (73.4)	324 (63.0)
	Important burden	421 (29.6)	235 (25.9)	186 (36.2)
	Missing	11 (0.8)	7 (0.8)	4 (0.8)
M ± SD		12.0 ± 23.0	10.5 ± 21.9	14.7 ± 24.5
**Pain** n (%)	No/low burden	744 (52.3)	477 (52.5)	267 (51.9)
	Important burden	672 (47.2)	428 (47.1)	244 (47.5)
	Missing	7 (0.6)	4 (0.4)	3 (0.6)
M ± SD		31.2 ± 34.0	30.5 ± 33.3	32.5 ± 35.2
**Dyspnoea** n (%)	No/low burden	727 (51.1)	460 (50.6)	267 (51.9)
	Important burden	684 (48.1)	442 (48.6)	242 (47.1)
	Missing	12 (0.8)	7 (0.8)	5 (1.0)
M ± SD		29.3 ± 35.2	29.5 ± 34.9	29.1 ± 35.9
**Insomnia** n (%)	No/low burden	945 (66.4)	640 (70.4)	305 (59.3)
	Important burden	469 (33.0)	263 (28.9)	206 (40.1)
	Missing	9 (0.6)	6 (0.7)	3 (0.6)
M ± SD		34.1 ± 37.1	31.2 ± 36.1	39.3 ± 38.3
**Appetite loss** n (%)	No/low burden	984 (69.1)	650 (71.5)	334 (65.0)
	Important burden	401 (28.2)	235 (25.9)	166 (32.3)
	Missing	38 (2.7)	24 (2.6)	14 (2.7)
M ± SD		29.3 ± 37.2	27.2 ± 36.6	33.0 ± 38.1
**Constipation** n (%)	No/low burden	1163 (81.7)	743 (81.7)	420 (81.7)
	Important burden	244 (17.1)	154 (16.9)	90 (17.5)
	Missing	16 (1.1)	12 (1.3)	4 (0.8)
M ± SD		17.8 ± 31.4	17.5 ± 31.1	18.4 ± 32.0
**Diarrhea** n (%)	No/low burden	1134 (79.7)	733 (80.6)	401 (78.0)
	Important burden	270 (19.0)	162 (17.8)	108 (21.0)
	Missing	19 (1.3)	14 (1.5)	5 (1.0)
M ± SD		10.7 ± 24.8	9.5 ± 22.7	12.9 ± 27.9
**Financhial difficulties** n (%)	No/low burden	966 (67.9)	606 (66.7)	360 (70.0)
	Important burden	417 (29.3)	276 (30.4)	141 (27.4)
	Missing	40 (2.8)	27 (3.0)	13 (2.5)
M ± SD		16.6 ± 29.0	16.9 ± 28.6	16.2 ± 29.6

### Subgroup analysis by age

Analysis of the functioning scales showed that, across all age groups, at least half of the patients reported a clinically important burden in physical functioning and, with the exception of the “very old” group, in emotional functioning. More than half of the participants in the two oldest groups reported a clinically important burden in role functioning, while more than half of the young adults reported a clinically important burden in social functioning. Regarding age-group patterns, physical functioning and - to a lesser extent, role functioning - showed a decline with increasing age, as reflected in both the percentages and the mean scores. Furthermore, both the percentages and mean scores indicate better social functioning with increasing age.

For symptom scales, across all age groups, at least half of the patients reported a clinically important burden for fatigue. For other scales, more than half of the participants reported a clinically important burden only in isolated subgroup-scale combinations, namely pain among middle-aged adults and dyspnea among the very old. Regarding age-group patterns, constipation and - when excluding young adults, appetite loss - showed a decline with increasing age, as reflected in both the percentages and the mean scores. Furthermore, both the percentages and mean scores indicate less financial problems with increasing age.

Among all scales, global health status showed the smallest difference in mean scores between age groups, within a 4-point range. Table 3 summarizes the analysis stratified by age.

**Table 3:** Age-stratified distribution according to Giesinger’s thresholds [19] and mean EORTC QLQ-C30 scale scores at inpatient radiation oncology admission (*n* = 1,423)

Functioning scales (higher scores are better)		Full sample *n* = 1423	Young adults (18–39) *n* = 21	Middle- aged adults (40–64) *n* = 615	Young-old (65–74) *n* = 427	Middle-old (75–84) *n* = 299	Very old (≥ 85) *n* = 61
**Physical functioning** n (%)	No/low burden	388 (27.3)	8 (38.1)	198 (32.2)	116 (27.2)	57 (19.1)	9 (14.8)
	Important burden	1033 (72.6)	13 (61.9)	415 (67.5)	311 (72.8)	242 (80.9)	52 (85.2)
	Missing	2 (0.1)	0 (0.0)	2 (0.3)	0 (0.0)	0 (0.0)	0 (0.0)
M ± SD		60.7 ± 29.1	69.7 ± 24.1	65.5 ± 27.6	60.3 ± 29.3	53.2 ± 29.4	47.9 ± 30.9
**Role functioning** n (%)	No/low burden	732 (51.4)	12 (57.1)	328 (53.3)	220 (51.5)	146 (48.8)	26 (42.6)
	Important burden	679 (47.7)	9 (42.9)	282 (45.9)	201 (47.1)	153 (51.2)	34 (55.7)
	Missing	12 (0.8)	0 (0.0)	5 (0.8)	6 (1.4)	0 (0.0)	1 (1.6)
M ± SD		56.7 ± 36.1	61.9 ± 33.4	58.8 ± 35.7	57.4 ± 35.9	51.8 ± 37.0	52.8 ± 35.8
**Emotional functioning** n (%)	No/low burden	646 (45.4)	9 (42.9)	274 (44.6)	201 (47.1)	134 (44.8)	28 (45.9)
	Important burden	759 (53.3)	12 (57.1)	338 (55.0)	218 (51.1)	161 (53.8)	30 (49.2)
	Missing	18 (1.3)	0 (0.0)	3 (0.5)	8 (1.9)	4 (1.3)	3 (4.9)
M ± SD		65.4 ± 25.4	61.6 ± 32.0	63.8 ± 25.9	67.8 ± 24.7	65.0 ± 24.9	69.1 ± 24.5
**Cognitive functioning** n (%)	No/low burden	930 (65.4)	11 (52.4)	406 (66.0)	282 (66.0)	197 (65.9)	34 (55.7)
	Important burden	478 (33.6)	10 (47.6)	206 (33.5)	138 (32.3)	100 (33.4)	24 (39.3)
	Missing	15 (1.1)	0 (0.0)	3 (0.5)	7 (1.6)	2 (0.7)	3 (4.9)
M ± SD		79.7 ± 25.5	79.4 ± 21.0	79.5 ± 26.0	80.7 ± 24.2	79.5 ± 26.4	75.0 ± 25.0
**Social functioning** n (%)	No/low burden	894 (62.8)	8 (38.1)	368 (59.8)	281 (65.8)	194 (64.9)	43 (70.5)
	Important burden	492 (34.6)	13 (61.9)	238 (38.7)	132 (30.9)	95 (31.8)	14 (23.0)
	Missing	37 (2.6)	0 (0.0)	9 (1.5)	14 (3.3)	10 (3.3)	4 (6.6)
M ± SD		65.6 ± 33.6	48.4 ± 35.7	61.7 ± 33.4	68.4 ± 33.7	68.6 ± 32.7	77.8 ± 31.7
**Global health status** M ± SD		49.6 ± 22.0	52.4 ± 23.7	48.8 ± 21.9	50.7 ± 22.6	49.7 ± 21.3	48.4 ± 21.9
**Symptom scales** (lower scores are better)							
**Fatigue** n (%)	No/low burden	667 (49.6)	8 (38.1)	297 (48.3)	217 (50.8)	124 (41.5)	21 (34.4)
	Important burden	747 (52.5)	13 (61.9)	315 (51.2)	206 (48.2)	175 (58.5)	38 (62.3)
	Missing	9 (0.6)	0 (0.0)	3 (0.5)	4 (0.9)	0 (0.0)	2 (3.3)
M ± SD		44.6 ± 29.9	55.0 ± 34.5	43.5 ± 30.3	42.8 ± 29.7	47.6 ± 28.8	51.0 ± 29.0
**Nausea and vomiting** n (%)	No/low burden	991 (69.6)	6 (28.6)	418 (68.0)	305 (71.4)	216 (72.2)	46 (75.4)
	Important burden	421 (29.6)	15 (71.4)	194 (31.5)	116 (27.2)	83 (27.8)	13 (21.3)
	Missing	11 (0.8)	0 (0.0)	3 (0.5)	6 (1.4)	0 (0.0)	2 (3.3)
M ± SD		12.0 ± 23.0	31.0 ± 29.5	13.0 ± 24.2	11.1 ± 22.3	10.1 ± 20.2	10.5 ± 22.5
**Pain** n (%)	No/low burden	744 (52.3)	11 (52.4)	294 (47.8)	234 (54.8)	173 (57.9)	32 (52.5)
	Important burden	672 (47.2)	10 (47.6)	320 (52.0)	188 (44.0)	126 (42.1)	28 (45.9)
	Missing	7 (0.6)	0 (0.0)	1 (0.2)	5 (1.2)	0 (0.0)	1 (1.6)
M ± SD		31.2 ± 34.0	35.7 ± 38.1	33.6 ± 33.8	29.5 ± 34.2	28.1 ± 33.0	32.5 ± 36.6
**Dyspnoea** n (%)	No/low burden	727 (51.1)	13 (61.9)	306 (49.8)	211 (49.4)	169 (56.5)	28 (45.9)
	Important burden	684 (48.1)	8 (38.1)	305 (49.6)	210 (49.2)	129 (43.1)	32 (52.5)
	Missing	12 (0.8)	0 (0.0)	4 (0.7)	6 (1.4)	1 (0.3)	1 (1.6)
M ± SD		29.3 ± 35.2	19.0 ± 29.0	29.1 ± 34.4	29.9 ± 35.1	28.6 ± 36.8	35.0 ± 39.0
**Insomnia** n (%)	No/low burden	945 (66.4)	14 (66.7)	396 (64.4)	301 (70.5)	196 (65.6)	38 (62.3)
	Important burden	469 (33.0)	7 (33.3)	217 (35.3)	120 (28.1)	103 (34.4)	22 (36.1)
	Missing	9 (0.6)	0 (0.0)	2 (0.3)	6 (1.4)	0 (0.0)	1 (1.6)
M ± SD		34.1 ± 37.1	30.2 ± 36.4	36.4 ± 37.3	30.1 ± 35.8	34.4 ± 38.2	38.9 ± 35.9
**Appetite loss** n (%)	No/low burden	984 (69.1)	13 (61.9)	442 (71.9)	293 (68.6)	197 (65.9)	39 (63.9)
	Important burden	401 (28.2)	8 (38.1)	159 (25.9)	120 (28.1)	94 (31.4)	20 (32.8)
	Missing	38 (2.7)	0 (0.0)	14 (2.3)	14 (3.3)	8 (2.7)	2 (3.3)
M ± SD		29.3 ± 37.2	34.9 ± 35.7	27.8 ± 35.7	28.7 ± 37.2	31.7 ± 39.6	34.5 ± 40.6
**Constipation** n (%)	No/low burden	1163 (81.7)	20 (95.2)	519 (84.4)	353 (82.7)	229 (76.6)	42 (68.9)
	Important burden	244 (17.1)	1 (4.8)	91 (14.8)	67 (15.7)	68 (22.7)	17 (27.9)
	Missing	16 (1.1)	0 (0.0)	5 (0.8)	7 (1.6)	2 (0.7)	2 (3.3)
M ± SD		17.8 ± 31.4	11.1 ± 19.2	14.9 ± 29.6	17.6 ± 31.0	23.2 ± 34.8	24.9 ± 34.2
**Diarrhea** n (%)	No/low burden	1134 (79.7)	14 (66.7)	484 (78.7)	351 (82.2)	240 (80.3)	45 (73.8)
	Important burden	270 (19.0)	7 (33.3)	123 (20.0)	69 (16.2)	57 (19.1)	14 (23.0)
	Missing	19 (1.3)	0 (0.0)	8 (1.3)	7 (1.6)	2 (0.7)	2 (3.3)
M ± SD		10.7 ± 24.8	17.5 ± 29.1	10.5 ± 23.4	9.7 ± 24.6	11.7 ± 26.8	13.6 ± 27.1
**Financhial difficulties** n (%)	No/low burden	966 (67.9)	8 (38.1)	334 (54.3)	329 (77.0)	244 (81.6)	51 (83.6)
	Important burden	417 (29.3)	13 (61.9)	265 (43.1)	85 (19.9)	48 (16.1)	6 (9.8)
	Missing	40 (2.8)	0 (0.0)	16 (2.6)	13 (3.0)	7 (2.3)	4 (6.6)
M ± SD		16.6 ± 29.0	38.1 ± 39.8	25.2 ± 33.2	10.4 ± 22.9	8.7 ± 22.1	4.7 ± 14.7

### Subgroup analysis by primary tumor

Primary tumors with more than 50 patients were head/neck (*n* = 422, 104 (24.6%) female; mean age 63.5 ± 10.0 years), lung (*n* = 398, 129 (32.4%) female; mean age 66.9 ± 10.1), colon/rectum (*n* = 127, 42 (33.1%) female; mean age 68.3 ± 11.0), upper GI tract (*n* = 86, 17 (19.8%) female; mean age 65.3 ± 11.0), and female genital organs (*n* = 77, 77 (100%) female; mean age 57.0 ± 15.2).

Analysis of the functioning scales showed that, across all diagnosis groups, more than half of the patients reported a clinically important burden for physical functioning. Emotional functioning showed the same tendency, except for the colon/rectum subgroup where less than half reported important burden. In role functioning, more than half of the lung subgroup reported a clinically important burden.

For the symptom scales, more than half of the lung subgroup reported a clinically important burden for fatigue and dyspnea, and more than half of the female genital organ subgroup for fatigue and nausea/vomiting.

The most visible diagnosis-specific patterns were a particularly high burden in physical and role functioning, fatigue, and dyspnoea in the lung subgroup; comparatively lower burden across most scales, except diarrhea, in the colon/rectum subgroup; and comparatively higher burden on most scales in the female genital organ subgroup. Among all diagnoses, global health status showed the smallest difference in mean scores between age groups, within a 2.9-point range. Table 4 summarizes the analysis stratified by primary tumor.

**Table 4:** Primary tumor-stratified distribution according to Giesinger’s thresholds [19] and mean EORTC QLQ-C30 scale scores at inpatient radiation oncology admission (*n* = 1,423)

Functioning scales (higher scores are better)		Full sample *n* = 1423	Head/neck *n* = 422	Lung *n* = 398	Colon/rectum *n* = 127	Upper GI tract *n* = 86	Female genital organs *n* = 77
**Physical functioning** n (%)	No/low burden	388 (27.3)	153 (36.3)	85 (21.4)	48 (37.8)	24 (27.9)	18 (23.4)
	Important burden	1033 (72.6)	269 (63.7)	313 (78.6)	79 (62.2)	62 (72.1)	59 (76.6)
	Missing	2 (0.1)	0 (0.0)	0 (0.0)	0 (0.0)	0 (0.0)	0 (0.0)
M ± SD		60.7 ± 29.1	69.4 ± 26.0	58.1 ± 26.8	68.5 ± 28.5	62.1 ± 26.7	59.4 ± 28.2
**Role functioning** n (%)	No/low burden	732 (51.4)	251 (59.5)	191 (48.0)	78 (61.4)	51 (59.3)	41 (53.2)
	Important burden	679 (47.7)	167 (39.6)	205 (51.5)	49 (38.6)	33 (38.4)	36 (46.8)
	Missing	12 (0.8)	4 (0.9)	2 (0.5)	0 (0.0)	2 (2.3)	0 (0.0)
M ± SD		56.7 ± 36.1	64.0 ± 33.5	53.9 ± 36.9	64.3 ± 33.5	63.9 ± 34.3	56.1 ± 34.6
**Emotional functioning** n (%)	No/low burden	646 (45.4)	202 (47.9)	175 (44.0)	71 (55.9)	39 (45.3)	28 (36.4)
	Important burden	759 (53.3)	215 (50.9)	221 (55.5)	53 (41.7)	46 (53.5)	48 (62.3)
	Missing	18 (1.3)	5 (1.2)	2 (0.5)	3 (2.4)	1 (1.2)	1 (1.3)
M ± SD		65.4 ± 25.4	67.2 ± 24.1	64.2 ± 25.7	72.0 ± 23.6	66.4 ± 25.6	60.3 ± 29.3
**Cognitive functioning** n (%)	No/low burden	930 (65.4)	299 (70.9)	249 (62.6)	97 (76.4)	60 (69.8)	54 (70.1)
	Important burden	478 (33.6)	118 (28.0)	148 (37.2)	27 (21.3)	25 (29.1)	23 (29.9)
	Missing	15 (1.1)	5 (1.2)	1 (0.3)	3 (2.4)	1 (1.2)	0 (0.0)
M ± SD		79.7 ± 25.5	84.1 ± 21.9	77.5 ± 26.2	86.2 ± 22.7	80.6 ± 25.4	81.0 ± 25.6
**Social functioning** n (%)	No/low burden	894 (62.8)	285 (67.5)	253 (63.6)	88 (69.3)	59 (68.6)	43 (55.8)
	Important burden	492 (34.6)	122 (28.9)	140 (35.2)	34 (26.8)	25 (29.1)	33 (42.9)
	Missing	37 (2.6)	15 (3.6)	5 (1.3)	5 (3.9)	2 (2.3)	1 (1.3)
M ± SD		65.6 ± 33.6	69.0 ± 30.6	65.9 ± 33.5	70.9 ± 31.5	68.1 ± 34.2	57.5 ± 37.2
**Global health status** M ± SD		49.6 ± 22.0	50.2 ± 20.8	50.1 ± 22.0	51.0 ± 23.6	50.6 ± 20.9	48.1 ± 22.0
**Symptom scales** (lower scores are better)							
**Fatigue** n (%)	No/low burden	667 (49.6)	238 (56.4)	168 (42.2)	73 (57.5)	43 (50.0)	26 (33.8)
	Important burden	747 (52.5)	180 (42.7)	230 (57.8)	52 (40.9)	42 (48.8)	51 (66.2)
	Missing	9 (0.6)	4 (0.9)	0 (0.0)	2 (1.6)	1 (1.2)	0 (0.0)
M ± SD		44.6 ± 29.9	37.7 ± 28.2	47.8 ± 29.6	35.4 ± 28.7	43.0 ± 27.2	54.0 ± 30.1
**Nausea and vomiting** n (%)	No/low burden	991 (69.6)	312 (73.9)	278 (69.8)	99 (78.0)	50 (58.1)	37 (48.1)
	Important burden	421 (29.6)	104 (24.6)	120 (30.2)	26 (20.5)	36 (41.9)	40 (51.9)
	Missing	11 (0.8)	6 (1.4)	0 (0.0)	2 (1.6)	0 (0.0)	0 (0.0)
M ± SD		12.0 ± 23.0	9.1 ± 19.7	11.9 ± 22.6	8.3 ± 20.5	14.0 ± 20.6	22.7 ± 29.1
**Pain** n (%)	No/low burden	744 (52.3)	225 (53.3)	209 (52.5)	74 (58.3)	44 (51.2)	39 (50.6)
	Important burden	672 (47.2)	194 (46.0)	189 (47.5)	52 (40.9)	41 (47.7)	38 (49.4)
	Missing	7 (0.6)	3 (0.7)	0 (0.0)	1 (0.8)	1 (1.2)	0 (0.0)
M ± SD		31.2 ± 34.0	28.3 ± 30.8	32.2 ± 35.6	26.1 ± 31.3	31.0 ± 32.8	32.9 ± 35.8
**Dyspnoea** n (%)	No/low burden	727 (51.1)	216 (51.2)	134 (33.7)	90 (70.9)	49 (57.0)	52 (67.5)
	Important burden	684 (48.1)	199 (47.2)	264 (66.3)	35 (27.6)	36 (41.9)	25 (32.5)
	Missing	12 (0.8)	7 (1.7)	0 (0.0)	2 (1.6)	1 (1.2)	0 (0.0)
M ± SD		29.3 ± 35.2	27.6 ± 33.6	43.2 ± 37.6	14.9 ± 26.9	23.1 ± 31.3	19.5 ± 31.7
**Insomnia** n (%)	No/low burden	945 (66.4)	295 (69.9)	245 (61.6)	96 (75.6)	54 (62.8)	47 (61.0)
	Important burden	469 (33.0)	123 (29.1)	153 (38.4)	29 (22.8)	31 (36.0)	30 (39.0)
	Missing	9 (0.6)	4 (0.9)	0 (0.0)	2 (1.6)	1 (1.2)	0 (0.0)
M ± SD		34.1 ± 37.1	31.5 ± 35.8	38.1 ± 38.2	25.6 ± 32.8	37.3 ± 38.3	38.1 ± 38.1
**Appetite loss** n (%)	No/low burden	984 (69.1)	298 (70.6)	278 (69.8)	89 (70.1)	54 (62.8)	47 (61.0)
	Important burden	401 (28.2)	98 (23.2)	117 (29.4)	36 (28.3)	29 (33.7)	30 (39.0)
	Missing	38 (2.7)	26 (6.2)	3 (0.8)	2 (1.6)	3 (3.5)	0 (0.0)
M ± SD		29.3 ± 37.2	26.6 ± 35.7	28.9 ± 37.1	28.8 ± 38.6	34.1 ± 38.9	36.8 ± 36.5
**Constipation** n (%)	No/low burden	1163 (81.7)	354 (83.9)	332 (83.4)	106 (83.5)	71 (82.6)	62 (80.5)
	Important burden	244 (17.1)	60 (14.2)	66 (16.6)	16 (12.6)	15 (17.4)	15 (19.5)
	Missing	16 (1.1)	8 (1.9)	0 (0.0)	5 (3.9)	0 (0.0)	0 (0.0)
M ± SD		17.8 ± 31.4	15.8 ± 29.1	16.8 ± 31.2	12.0 ± 28.4	18.2 ± 33.0	20.8 ± 32.5
**Diarrhea** n (%)	No/low burden	1134 (79.7)	348 (82.5)	346 (86.9)	76 (59.8)	68 (79.1)	45 (58.4)
	Important burden	270 (19.0)	66 (15.6)	51 (12.8)	46 (36.2)	18 (20.9)	31 (40.3)
	Missing	19 (1.3)	8 (1.9)	1 (0.3)	5 (3.9)	0 (0.0)	1 (1.3)
M ± SD		10.7 ± 24.8	8.1 ± 20.6	6.5 ± 19.6	21.9 ± 32.0	13.2 ± 29.0	24.6 ± 34.2
**Financhial difficulties** n (%)	No/low burden	966 (67.9)	264 (62.6)	280 (70.4)	91 (71.7)	55 (64.0)	38 (49.4)
	Important burden	417 (29.3)	142 (33.6)	114 (28.6)	31 (24.4)	28 (32.6)	37 (48.1)
	Missing	40 (2.8)	16 (3.8)	4 (1.0)	5 (3.9)	3 (3.5)	2 (2.6)
M ± SD		16.6 ± 29.0	18.5 ± 29.0	16.8 ± 30.0	13.9 ± 27.7	17.3 ± 27.2	29.8 ± 36.6

The supplementary materials includes results of all analyses with an added third category to enhance clinical interpretation.

## Discussio

Unlike most studies that focus on specific tumor entities or treatments, this analysis captures the full diagnostic and therapeutic spectrum seen in real-world inpatient radiation oncology. Robust PRO datasets collected under routine care remain rare because PRO assessment is not yet widely implemented in practice [7, 14, 15]. Routine PRO assessment not only enhances individual care but also allows for analysis of aggregated, real-world PRO data that reflect patient and treatment heterogeneity, helping to define population-level and setting-specific priorities. Identifying functions and symptoms with particularly severe burden can inform targeted resource allocation, such as supportive therapies and clinical algorithms.

The ePRO identified substantial self-perceived burden at inpatient radiotherapy (RT) admission, with more than half of patients reporting clinically important burden in physical and emotional functioning and fatigue. A prior study comparing electronically assessed EORTC QLQ-C30 data with standard clinical documentation at RT admission demonstrated that ePRO detected nearly three times as many symptoms, underscoring the value of PROs in identifying unmet patient needs [17]. Assessing quality of life at admission can inform treatment decisions and prompt early preventive and supportive interventions to avoid complications. However, merely collecting PRO data is insufficient to improve patient outcomes [23]. To maximize the potential of PRO assessment, data must be reviewed with patients and HCP to define care priorities and guide tailored oncologic treatment. In the clinic where the implementation took place, PRO-based symptom-specific recommendations were developed, including supportive care interventions and patient self-management guidance [17].

### Subgroup analyses

The sex-stratified analysis showed that physical and emotional functioning, as well as fatigue, were rated as burdensome by a majority in both groups. Overall and consistent with prior oncology studies, male patients reported better functioning and lower symptom burden than female patients [24]. However, German population norm data for the EORTC QLQ-C30 show similar sex differences [25], suggesting that these disparities may not be cancer-specific but rather reflect general differences in symptom reporting. For example, men may downplay symptoms due to perceived social expectations of masculinity [26]. This disparity between male and female burden ratings may also, at least partly, explain the higher reported burden in the female genital organ subgroup compared with other diagnosis-specific subgroups, in which men were the majority. While the largest mean differences were observed for less observable problems (emotional burden, fatigue, and insomnia), the overall magnitude of the mean differences ranged from trivial to small at most, according to the interpretation guidelines proposed by Cocks [27], indicating little, if any, clinical relevance, at least at the group level.

The age-stratified analysis demonstrated that physical and emotional functioning, along with fatigue, were rated as burdensome by a majority in most age groups, mirroring findings from the sex-stratified analysis. Self-perceived physical functioning decreased with older age, while appetite loss and constipation became more pronounced. While age-related decline in physical functioning aligns with German population norm data [25], the observed increases in constipation and appetite loss do not. This suggests that these symptoms may be exacerbated by age-specific effects of cancer or its treatment, rather than being solely attributable to normal aging. In contrast, older patients reported better social functioning and fewer financial difficulties. Compared to general population data [25], where age-related differences in these domains are minimal, our findings indicate a disproportionate burden on younger adults. This may reflect the greater disruption cancer and its treatment impose on their lives, such as impacts on social networks, career stability, and income dependency. In comparison, older patients may have fewer social contacts to be disrupted and a more stable financial status not reliant on wages.

Results for the most common tumor diagnoses were consistent with previously reported tumor-specific symptom patterns, such as severe dyspnea in lung cancer [28] and pronounced diarrhea in rectal cancer [29]. Compared with sex and age subgroups, ratings, especially on the symptom scales, were less consistent across diagnosis groups, suggesting greater heterogeneity in symptom experiences by diagnosis. Patients with lung cancer reported particularly high burden in physical and role functioning, fatigue, and dyspnea, largely consistent with another German study using the EORTC QLQ-C30 in patients with lung cancer [30]. Patients with colorectal cancer reported comparatively lower burden on most scales. The mean EORTC QLQ-C30 scale scores are consistent with those of another German study of rectal cancer patients undergoing neoadjuvant therapy [31].

While Giesinger et al.‘s thresholds can effectively categorize EORTC QLQ-C30 data, their dichotomous nature may limit practical utility when being used as a basis for color coding. Consequently, the color coding should not be interpreted independently of the corresponding scale scores, since substantial differences in scores may be represented by the same visual burden category, whereas minor score differences may be depicted as entirely different burden levels.

To address this, our clinic implemented a three-tier color-coding system (green/yellow/red) that combines Giesinger’s thresholds with EORTC QLQ-C30 data from an Austrian population norm study [32]. These data were available to the software provider and are based on a population considered sufficiently comparable to the German population. Although this third category lacks the scientific rigor of Giesinger’s thresholds and is not derived from German EORTC QLQ-C30 population norm data [25] - which were not available in a format that would permit the required percentile-based calculations - it facilitates a more nuanced graphical interpretation while preserving the thresholds as the primary cutoffs. The supplementary material demonstrates this tripartite application, showing how it influences visual display and clinical interpretability of analysis results.

Across all stratifications by sex, age, and tumor diagnosis, the global health status scale showed minimal variation between subgroups, maintaining scores around 50.0 (± 4.0) on the EORTC QLQ-C30’s 0-100 scale. This consistency likely reflects response shift [33], whereby patients recalibrate their internal standards and redefine “normal” health as their circumstances change. Despite reported differences in specific functional and symptom scales, this adaptive process may lead patients to rate their overall HRQOL consistently.

### Limitations

Only 25% of admitted patients completed the ePRO at admission between February 25, 2019, and June 14, 2024, markedly lower than the 60.8–78.8% rates reported during dedicated implementation studies [34, 35]. This completion rate highlights the distinct challenges of implementing ePRO in routine clinical care versus controlled research settings. Resource constraints necessitated using dedicated student assistants for ePRO introduction, who could only provide limited coverage (1–2 h daily with irregular schedules), leaving many admitted patients without assessment opportunities. As their availability patterns were non-systematic, we assume that increased participation would maintain the demographic and diagnostic distributions observed in our sample. To improve completion rates, all newly admitted patients should be approached, e.g., by having continuously available nursing staff introduce the assessment and assist with completion. The COVID-19 pandemic presented another barrier, causing several months of complete ePRO suspension. Because the student assistants collected ePRO data as part of routine clinical care rather than for research, no non-responder analysis was conducted. However, the previously cited ePRO implementation study at this clinic found that the main reasons for non-completion were lack of motivation or perceived benefit, forgetting, and temporary factors such as family visits, upcoming therapy, or high burden [17].

The analysis was limited to stratification by sex, age, and tumor diagnosis, despite patients presenting with varying tumor stages, treatment phases, and modalities that may influence symptom burden. Further differentiation would have created samples too small to analyze meaningfully.

Future research should leverage multi-center PRO datasets from comparable settings to achieve sufficient sample sizes for stratification by tumor stage and treatment characteristics alongside demographic factors. This would enable more precise identification of setting-specific care priorities. Additionally, analyzing characteristics of non-participants could provide critical insights into PRO assessment limitations and inform strategies to improve completion rates.

## Conclusion

Findings indicate high self-reported burden in patients at radiotherapy admission, highlighting the value of ePRO in identifying critical needs and the importance of early supportive care. Effective PRO assessment should combine specific scales (e.g., symptoms) for targeted measurement of relevant issues with global quality-of-life questions to include individual concerns beyond standardized formats. Successful clinical integration can be facilitated by developing balanced categorization systems to interpret PRO data, detailed enough to detect meaningful clinical differences while remaining simple enough to allow a differentiated reaction for each category. Aggregated real-world PRO data can inform care priorities and guide resource allocation for specific patient groups, requiring consistent data collection procedures and high participation rates. In this regard, expanding ePRO implementation across oncology settings would enable more detailed analyses through larger datasets.

## Electronic Supplementary Material

Below is the link to the electronic supplementary material.

Supplementary Material

## Data Availability

The datasets used and/or analysed within the project are available from the corresponding author on reasonable request.

## Abbreviations

GI: Gastrointestinal
HCP: Healthcare professionals
HRQOL: Health-related quality of life
M: Mean
PRO: Patient-reported outcomes
PROM: Patient-reported outcomes measures
QoL: Quality of life
RT: Radiotherapy
SD: Standard deviation
WHO: World health organization
