# Supplementary material for: Electronic patient-reported outcome assessment at inpatient admission: a descriptive analysis of real-world data for 1423 radiotherapy patients
Source: Radiat Oncol. 2026 Oct 23;21:141. doi: 10.1186/s13014-026-02947-1 (PMC13695275; doi:10.1186/s13014-026-02947-1)
Supplement: Supplementary file 1 [file 13014_2026_2947_MOESM1_ESM.docx]

**Supplementary material**

Sex-stratified distribution based on tripartite [19, 32] and mean EORTC QLQ-C30 scale scores at inpatient radiation oncology admission (n = 1,423)

| **Functioning scales**  (higher scores are better) | | **Full**  **sample**  n = 1423 | **Male**  n = 909 | **Female**  n = 514 |
| --- | --- | --- | --- | --- |
| **Physical functioning**  n (%) | No/low burden | 388 (27.3) | 267 (29.4) | 121 (23.5) |
|  | Moderate burden | 339 (23.8) | 224 (24.6) | 115 (22.4) |
|  | Important burden | 694 (48.8) | 416 (45.8) | 278 (54.1) |
|  | Missing | 2 (0.1) | 2 (0.2) | 0 (0.0) |
| M±SD | | 60.7±29.1 | 62.5±28.8 | 57.4±29.2 |
| **Role functioning**  n (%) | No/low burden | 732 (51.4) | 480 (52.8) | 252 (49.0) |
|  | Moderate burden | 353 (24.8) | 219 (24.1) | 134 (26.1) |
|  | Important burden | 326 (22.9) | 202 (22.2) | 124 (24.1) |
|  | Missing | 12 (0.8) | 8 (0.9) | 4 (0.8) |
| M±SD | | 56.7±36.1 | 58.1±36.3 | 54.2±35.6 |
| **Emotional functioning**  n (%) | No/low burden | 646 (45.4) | 459 (50.5) | 187 (36.4) |
|  | Moderate burden | 429 (30.1) | 266 (29.3) | 163 (31.7) |
|  | Important burden | 330 (23.2) | 172 (18.9) | 158 (30.7) |
|  | Missing | 18 (1.3) | 12 (1.3) | 6 (1.2) |
| M±SD | | 65.4±25.4 | 68.3±24.1 | 60.3±26.9 |
| **Cognitive functioning**  n (%) | No/low burden | 930 (65.4) | 617 (67.9) | 313 (60.9) |
|  | Moderate burden | 342 (24.0) | 207 (22.8) | 135 (26.3) |
|  | Important burden | 136 (9.6) | 75 (8.3) | 61 (11.9) |
|  | Missing | 15 (1.1) | 10 (1.1) | 5 (1.0) |
| M±SD | | 79.7±25.5 | 81.3±24.0 | 76.8±27.6 |
| **Social functioning**  n (%) | No/low burden | 894 (62.8) | 579 (63.7) | 315 (61.3) |
|  | Moderate burden | 273 (19.2) | 175 (19.3) | 98 (19.1) |
|  | Important burden | 219 (15.4) | 132 (14.5) | 87 (16.9) |
|  | Missing | 37 (2.6) | 23 (2.5) | 14 (2.7) |
| M±SD | | 65.6±33.6 | 66.1±32.9 | 64.7±34.7 |
| **Global health status** M±SD | | 49.6±22.0 | 49.4±21.2 | 50.0±23.4 |
| **Symptom scales**  (lower scores are better) | |  |  |  |
| **Fatigue**  n (%) | No/low burden | 300 (21.1) | 214 (23.5) | 86 (16.7) |
|  | Moderate burden | 367 (25.8) | 258 (28.4) | 109 (21.2) |
|  | Important burden | 747 (52.5) | 431 (47.4) | 316 (61.5) |
|  | Missing | 9 (0.6) | 6 (0.7) | 3 (0.6) |
| M±SD | | 44.6±29.9 | 41.4±29.6 | 50.3±29.6 |
| **Nausea and vomiting**  n (%) | No/low burden | 991 (69.6) | 667 (73.4) | 324 (63.0) |
|  | Moderate burden | - | - | - |
|  | Important burden | 421 (29.6) | 235 (25.9) | 186 (36.2) |
|  | Missing | 11 (0.8) | 7 (0.8) | 4 (0.8) |
| M±SD | | 12.0±23.0 | 10.5±21.9 | 14.7±24.5 |
| **Pain**  n (%) | No/low burden | 595 (41.8) | 383 (42.1) | 212 (41.2) |
|  | Moderate burden | 149 (10.5) | 94 (10.3) | 55 (10.7) |
|  | Important burden | 672 (47.2) | 428 (47.1) | 244 (47.5) |
|  | Missing | 7 (0.6) | 4 (0.4) | 3 (0.6) |
| M±SD | | 31.2±34.0 | 30.5±33.3 | 32.5±35.2 |
| **Dyspnoea**  n (%) | No/low burden | 727 (51.1) | 460 (50.6) | 267 (51.9) |
|  | Moderate burden | - | - | - |
|  | Important burden | 684 (48.1) | 442 (48.6) | 242 (47.1) |
|  | Missing | 12 (0.8) | 7 (0.8) | 5 (1.0) |
| M±SD | | 29.3±35.2 | 29.5±34.9 | 29.1±35.9 |
| **Insomnia**  n (%) | No/low burden | 646 (45.4) | 439 (48.3) | 207 (40.3) |
|  | Moderate burden | 299 (21.0) | 201 (22.1) | 98 (19.1) |
|  | Important burden | 469 (33.0) | 263 (28.9) | 206 (40.1) |
|  | Missing | 9 (0.6) | 6 (0.7) | 3 (0.6) |
| M±SD | | 34.1±37.1 | 31.2±36.1 | 39.3±38.3 |
| **Appetite loss**  n (%) | No/low burden | 764 (53.7) | 514 (56.5) | 250 (48.6) |
|  | Moderate burden | 220 (15.5) | 136 (15.0) | 84 (16.3) |
|  | Important burden | 401 (28.2) | 235 (25.9) | 166 (32.3) |
|  | Missing | 38 (2.7) | 24 (2.6) | 14 (2.7) |
| M±SD | | 29.3±37.2 | 27.2±36.6 | 33.0±38.1 |
| **Constipation**  n (%) | No/low burden | 1001 (70.3) | 642 (70.6) | 359 (69.8) |
|  | Moderate burden | 162 (11.4) | 101 (11.1) | 61 (11.9) |
|  | Important burden | 244 (17.1) | 154 (16.9) | 90 (17.5) |
|  | Missing | 16 (1.1) | 12 (1.3) | 4 (0.8) |
| M±SD | | 17.8±31.4 | 17.5±31.1 | 18.4±32.0 |
| **Diarrhea**  n (%) | No/low burden | 1134 (79.7) | 733 (80.6) | 401 (78.0) |
|  | Moderate burden | - | - | - |
|  | Important burden | 270 (19.0) | 162 (17.8) | 108 (21.0) |
|  | Missing | 19 (1.3) | 14 (1.5) | 5 (1.0) |
| M±SD | | 10.7±24.8 | 9.5±22.7 | 12.9±27.9 |
| **Financial difficulties**  n (%) | No/low burden | 966 (67.9) | 606 (66.7) | 360 (70.0) |
|  | Moderate burden | - | - | - |
|  | Important burden | 417 (29.3) | 276 (30.4) | 141 (27.4) |
|  | Missing | 40 (2.8) | 27 (3.0) | 13 (2.5) |
| M±SD | | 16.6±29.0 | 16.9±28.6 | 16.2±29.6 |

- Threshold too low to calculate third category

Age-stratified distribution based on tripartite [19, 32] and mean EORTC QLQ-C30 scale scores at inpatient radiation oncology admission (n = 1,423)

| **Functioning scales**  (higher scores are better) | | **Full**  **sample**  n = 1423 | **Young adults**  (18-39)  n = 21 | **Middle-**  **aged**  **adults**  (40-64)  n = 615 | **Young-old**  (65-74)  n = 427 | **Middle-old**  (75-84)  n = 299 | **Very-old**  (≥85)  n = 61 |
| --- | --- | --- | --- | --- | --- | --- | --- |
| **Physical functioning**  n (%) | No/low burden | 388 (27.3) | 8 (38.1) | 198 (32.2) | 116 (27.2) | 57 (19.1) | 9 (14.8) |
|  | Moderate burden | 339 (23.8) | 4 (19.0) | 164 (26.7) | 96 (22.5) | 59 (19.7) | 16 (26.2) |
|  | Important burden | 694 (48.8) | 9 (42.9) | 251 (40.8) | 215 (50.4) | 183 (61.2) | 36 (59.0) |
|  | Missing | 2 (0.1) | 0 (0.0) | 2 (0.3) | 0 (0.0) | 0 (0.0) | 0 (0.0) |
| M±SD | | 60.7±29.1 | 69.7±24.1 | 65.5±27.6 | 60.3±29.3 | 53.2±29.4 | 47.9±30.9 |
| **Role functioning**  n (%) | No/low burden | 732 (51.4) | 12 (57.1) | 328 (53.3) | 220 (51.5) | 146 (48.8) | 26 (42.6) |
|  | Moderate burden | 353 (24.8) | 5 (23.8) | 158 (25.7) | 109 (25.5) | 62 (20.7) | 19 (31.1) |
|  | Important burden | 326 (22.9) | 4 (19.0) | 124 (20.2) | 92 (21.5) | 91 (30.4) | 15 (24.6) |
|  | Missing | 12 (0.8) | 0 (0.0) | 5 (0.8) | 6 (1.4) | 0 (0.0) | 1 (1.6) |
| M±SD | | 56.7±36.1 | 61.9±33.4 | 58.8±35.7 | 57.4±35.9 | 51.8±37.0 | 52.8±35.8 |
| **Emotional functioning**  n (%) | No/low burden | 646 (45.4) | 9 (42.9) | 274 (44.6) | 201 (47.1) | 134 (44.8) | 28 (45.9) |
|  | Moderate burden | 429 (30.1) | 5 (23.8) | 186 (30.2) | 135 (31.6) | 85 (28.4) | 18 (29.5) |
|  | Important burden | 330 (23.2) | 7 (33.3) | 152 (24.7) | 83 (19.4) | 76 (25.4) | 12 (19.7) |
|  | Missing | 18 (1.3) | 0 (0.0) | 3 (0.5) | 8 (1.9) | 4 (1.3) | 3 (4.9) |
| M±SD | | 65.4±25.4 | 61.6±32.0 | 63.8±25.9 | 67.8±24.7 | 65.0±24.9 | 69.1±24.5 |
| **Cognitive functioning**  n (%) | No/low burden | 930 (65.4) | 11 (52.4) | 406 (66.0) | 282 (66.0) | 197 (65.9) | 34 (55.7) |
|  | Moderate burden | 342 (24.0) | 9 (42.9) | 146 (23.7) | 104 (24.4) | 66 (22.1) | 17 (27.9) |
|  | Important burden | 136 (9.6) | 1 (4.8) | 60 (9.8) | 34 (8.0) | 34 (11.4) | 7 (11.5) |
|  | Missing | 15 (1.1) | 0 (0.0) | 3 (0.5) | 7 (1.6) | 2 (0.7) | 3 (4.9) |
| M±SD | | 79.7±25.5 | 79.4±21.0 | 79.5±26.0 | 80.7±24.2 | 79.5±26.4 | 75.0±25.0 |
| **Social functioning**  n (%) | No/low burden | 894 (62.8) | 8 (38.1) | 368 (59.8) | 281 (65.8) | 194 (64.9) | 43 (70.5) |
|  | Moderate burden | 273 (19.2) | 6 (28.6) | 130 (21.1) | 72 (16.9) | 56 (18.7) | 9 (14.8) |
|  | Important burden | 219 (15.4) | 7 (33.3) | 108 (17.6) | 60 (14.1) | 39 (13.0) | 5 (8.2) |
|  | Missing | 37 (2.6) | 0 (0.0) | 9 (1.5) | 14 (3.3) | 10 (3.3) | 4 (6.6) |
| M±SD | | 65.6±33.6 | 48.4±35.7 | 61.7±33.4 | 68.4±33.7 | 68.6±32.7 | 77.8±31.7 |
| **Global health status** M±SD | | 49.6±22.0 | 52.4±23.7 | 48.8±21.9 | 50.7±22.6 | 49.7±21.3 | 48.4±21.9 |
| **Symptom scales**  (lower scores are better) | |  |  |  |  |  |  |
| **Fatigue**  n (%) | No/low burden | 300 (21.1) | 4 (19.0) | 144 (23.4) | 90 (21.1) | 54 (18.1) | 8 (13.1) |
|  | Moderate burden | 367 (25.8) | 4 (19.0) | 153 (24.9) | 127 (29.7) | 70 (23.4) | 13 (21.3) |
|  | Important burden | 747 (52.5) | 13 (61.9) | 315 (51.2) | 206 (48.2) | 175 (58.5) | 38 (62.3) |
|  | Missing | 9 (0.6) | 0 (0.0) | 3 (0.5) | 4 (0.9) | 0 (0.0) | 2 (3.3) |
| M±SD | | 44.6±29.9 | 55.0±34.5 | 43.5±30.3 | 42.8±29.7 | 47.6±28.8 | 51.0±29.0 |
| **Nausea and vomiting**  n (%) | No/low burden | 991 (69.6) | 6 (28.6) | 418 (68.0) | 305 (71.4) | 216 (72.2) | 46 (75.4) |
|  | Moderate burden | - | – | – | – | – | – |
|  | Important burden | 421 (29.6) | 15 (71.4) | 194 (31.5) | 116 (27.2) | 83 (27.8) | 13 (21.3) |
|  | Missing | 11 (0.8) | 0 (0.0) | 3 (0.5) | 6 (1.4) | 0 (0.0) | 2 (3.3) |
| M±SD | | 12.0±23.0 | 31.0±29.5 | 13.0±24.2 | 11.1±22.3 | 10.1±20.2 | 10.5±22.5 |
| **Pain**  n (%) | No/low burden | 595 (41.8) | 7 (33.3) | 234 (38.0) | 191 (44.7) | 136 (45.5) | 27 (44.3) |
|  | Moderate burden | 149 (10.5) | 4 (19.0) | 60 (9.8) | 43 (10.1) | 37 (12.4) | 5 (8.2) |
|  | Important burden | 672 (47.2) | 10 (47.6) | 320 (52.0) | 188 (44.0) | 126 (42.1) | 28 (45.9) |
|  | Missing | 7 (0.6) | 0 (0.0) | 1 (0.2) | 5 (1.2) | 0 (0.0) | 1 (1.6) |
| M±SD | | 31.2±34.0 | 35.7±38.1 | 33.6±33.8 | 29.5±34.2 | 28.1±33.0 | 32.5±36.6 |
| **Dyspnoea**  n (%) | No/low burden | 727 (51.1) | 13 (61.9) | 306 (49.8) | 211 (49.4) | 169 (56.5) | 28 (45.9) |
|  | Moderate burden | - | – | – | – | – | – |
|  | Important burden | 684 (48.1) | 8 (38.1) | 305 (49.6) | 210 (49.2) | 129 (43.1) | 32 (52.5) |
|  | Missing | 12 (0.8) | 0 (0.0) | 4 (0.7) | 6 (1.4) | 1 (0.3) | 1 (1.6) |
| M±SD | | 29.3±35.2 | 19.0±29.0 | 29.1±34.4 | 29.9±35.1 | 28.6±36.8 | 35.0±39.0 |
| **Insomnia**  n (%) | No/low burden | 646 (45.4) | 11 (52.4) | 259 (42.1) | 213 (49.9) | 142 (47.5) | 21 (34.4) |
|  | Moderate burden | 299 (21.0) | 3 (14.3) | 137 (22.3) | 88 (20.6) | 54 (18.1) | 17 (27.9) |
|  | Important burden | 469 (33.0) | 7 (33.3) | 217 (35.3) | 120 (28.1) | 103 (34.4) | 22 (36.1) |
|  | Missing | 9 (0.6) | 0 (0.0) | 2 (0.3) | 6 (1.4) | 0 (0.0) | 1 (1.6) |
| M±SD | | 34.1±37.1 | 30.2±36.4 | 36.4±37.3 | 30.1±35.8 | 34.4±38.2 | 38.9±35.9 |
| **Appetite loss**  n (%) | No/low burden | 764 (53.7) | 9 (42.9) | 331 (53.8) | 234 (54.8) | 160 (53.5) | 30 (49.2) |
|  | Moderate burden | 220 (15.5) | 4 (19.0) | 111 (18.0) | 59 (13.8) | 37 (12.4) | 9 (14.8) |
|  | Important burden | 401 (28.2) | 8 (38.1) | 159 (25.9) | 120 (28.1) | 94 (31.4) | 20 (32.8) |
|  | Missing | 38 (2.7) | 0 (0.0) | 14 (2.3) | 14 (3.3) | 8 (2.7) | 2 (3.3) |
| M±SD | | 29.3±37.2 | 34.9±35.7 | 27.8±35.7 | 28.7±37.2 | 31.7±39.6 | 34.5±40.6 |
| **Constipation**  n (%) | No/low burden | 1001 (70.3) | 15 (71.4) | 465 (75.6) | 296 (69.3) | 189 (63.2) | 36 (59.0) |
|  | Moderate burden | 162 (11.4) | 5 (23.8) | 54 (8.8) | 57 (13.3) | 40 (13.4) | 6 (9.8) |
|  | Important burden | 244 (17.1) | 1 (4.8) | 91 (14.8) | 67 (15.7) | 68 (22.7) | 17 (27.9) |
|  | Missing | 16 (1.1) | 0 (0.0) | 5 (0.8) | 7 (1.6) | 2 (0.7) | 2 (3.3) |
| M±SD | | 17.8±31.4 | 11.1±19.2 | 14.9±29.6 | 17.6±31.0 | 23.2±34.8 | 24.9±34.2 |
| **Diarrhea**  n (%) | No/low burden | 1134 (79.7) | 14 (66.7) | 484 (78.7) | 351 (82.2) | 240 (80.3) | 45 (73.8) |
|  | Moderate burden | - | – | – | – | – | – |
|  | Important burden | 270 (19.0) | 7 (33.3) | 123 (20.0) | 69 (16.2) | 57 (19.1) | 14 (23.0) |
|  | Missing | 19 (1.3) | 0 (0.0) | 8 (1.3) | 7 (1.6) | 2 (0.7) | 2 (3.3) |
| M±SD | | 10.7±24.8 | 17.5±29.1 | 10.5±23.4 | 9.7±24.6 | 11.7±26.8 | 13.6±27.1 |
| **Financial difficulties**  n (%) | No/low burden | 966 (67.9) | 8 (38.1) | 334 (54.3) | 329 (77.0) | 244 (81.6) | 51 (83.6) |
|  | Moderate burden | - | – | – | – | – | – |
|  | Important burden | 417 (29.3) | 13 (61.9) | 265 (43.1) | 85 (19.9) | 48 (16.1) | 6 (9.8) |
|  | Missing | 40 (2.8) | 0 (0.0) | 16 (2.6) | 13 (3.0) | 7 (2.3) | 4 (6.6) |
| M±SD | | 16.6±29.0 | 38.1±39.8 | 25.2±33.2 | 10.4±22.9 | 8.7±22.1 | 4.7±14.7 |

- Threshold too low to calculate third category

Primary tumor-stratified distribution based on tripartite [19, 32] and mean EORTC QLQ-C30 scale scores at inpatient radiation oncology admission (n = 1,423)

| **Functioning scales**  (higher scores are better) | | **Full**  **sample**  n = 1423 | **Head/ neck**  n = 422 | **Lung**  n = 398 | **Colon/ rectum**  n = 127 | **Upper GI tract**  n = 86 | **Female genital organs**  n = 77 |
| --- | --- | --- | --- | --- | --- | --- | --- |
| **Physical functioning**  n (%) | No/low burden | 388 (27.3) | 153 (36.3) | 85 (21.4) | 48 (37.8) | 24 (27.9) | 18 (23.4) |
|  | Moderate burden | 339 (23.8) | 118 (28.0) | 91 (22.9) | 33 (26.0) | 22 (25.6) | 20 (26.0) |
|  | Important burden | 694 (48.8) | 151 (35.8) | 222 (55.8) | 46 (36.2) | 40 (46.5) | 39 (50.6) |
|  | Missing | 2 (0.1) | 0 (0.0) | 0 (0.0) | 0 (0.0) | 0 (0.0) | 0 (0.0) |
| M±SD | | 60.7±29.1 | 69.4±26.0 | 58.1±26.8 | 68.5±28.5 | 62.1±26.7 | 59.4±28.2 |
| **Role functioning**  n (%) | No/low burden | 732 (51.4) | 251 (59.5) | 191 (48.0) | 78 (61.4) | 51 (59.3) | 41 (53.2) |
|  | Moderate burden | 353 (24.8) | 105 (24.9) | 102 (25.6) | 30 (23.6) | 20 (23.3) | 19 (24.7) |
|  | Important burden | 326 (22.9) | 62 (14.7) | 103 (25.9) | 19 (15.0) | 13 (15.1) | 17 (22.1) |
|  | Missing | 12 (0.8) | 4 (0.9) | 2 (0.5) | 0 (0.0) | 2 (2.3) | 0 (0.0) |
| M±SD | | 56.7±36.1 | 64.0±33.5 | 53.9±36.9 | 64.3±33.5 | 63.9±34.3 | 56.1±34.6 |
| **Emotional functioning**  n (%) | No/low burden | 646 (45.4) | 202 (47.9) | 175 (44.0) | 71 (55.9) | 39 (45.3) | 28 (36.4) |
|  | Moderate burden | 429 (30.1) | 129 (30.6) | 118 (29.6) | 34 (26.8) | 28 (32.6) | 24 (31.2) |
|  | Important burden | 330 (23.2) | 86 (20.4) | 103 (25.9) | 19 (15.0) | 18 (20.9) | 24 (31.2) |
|  | Missing | 18 (1.3) | 5 (1.2) | 2 (0.5) | 3 (2.4) | 1 (1.2) | 1 (1.3) |
| M±SD | | 65.4±25.4 | 67.2±24.1 | 64.2±25.7 | 72.0±23.6 | 66.4±25.6 | 60.3±29.3 |
| **Cognitive functioning**  n (%) | No/low burden | 930 (65.4) | 299 (70.9) | 249 (62.6) | 97 (76.4) | 60 (69.8) | 54 (70.1) |
|  | Moderate burden | 342 (24.0) | 94 (22.3) | 106 (26.6) | 18 (14.2) | 16 (18.6) | 16 (20.8) |
|  | Important burden | 136 (9.6) | 24 (5.7) | 42 (10.6) | 9 (7.1) | 9 (10.5) | 7 (9.1) |
|  | Missing | 15 (1.1) | 5 (1.2) | 1 (0.3) | 3 (2.4) | 1 (1.2) | 0 (0.0) |
| M±SD | | 79.7±25.5 | 84.1±21.9 | 77.5±26.2 | 86.2±22.7 | 80.6±25.4 | 81.0±25.6 |
| **Social functioning**  n (%) | No/low burden | 894 (62.8) | 285 (67.5) | 253 (63.6) | 88 (69.3) | 59 (68.6) | 43 (55.8) |
|  | Moderate burden | 273 (19.2) | 80 (19.0) | 79 (19.8) | 19 (15.0) | 10 (11.6) | 15 (19.5) |
|  | Important burden | 219 (15.4) | 42 (10.0) | 61 (15.3) | 15 (11.8) | 15 (17.4) | 18 (23.4) |
|  | Missing | 37 (2.6) | 15 (3.6) | 5 (1.3) | 5 (3.9) | 2 (2.3) | 1 (1.3) |
| M±SD | | 65.6±33.6 | 69.0±30.6 | 65.9±33.5 | 70.9±31.5 | 68.1±34.2 | 57.5±37.2 |
| **Global health status** M±SD | | 49.6±22.0 | 50.2±20.8 | 50.1±22.0 | 51.0±23.6 | 50.6±20.9 | 48.1±22.0 |
| **Symptom scales**  (lower scores are better) | |  |  |  |  |  |  |
| **Fatigue**  n (%) | No/low burden | 300 (21.1) | 118 (28.0) | 69 (17.3) | 40 (31.5) | 15 (17.4) | 10 (13.0) |
|  | Moderate burden | 367 (25.8) | 120 (28.4) | 99 (24.9) | 33 (26.0) | 28 (32.6) | 16 (20.8) |
|  | Important burden | 747 (52.5) | 180 (42.7) | 230 (57.8) | 52 (40.9) | 42 (48.8) | 51 (66.2) |
|  | Missing | 9 (0.6) | 4 (0.9) | 0 (0.0) | 2 (1.6) | 1 (1.2) | 0 (0.0) |
| M±SD | | 44.6±29.9 | 37.7±28.2 | 47.8±29.6 | 35.4±28.7 | 43.0±27.2 | 54.0±30.1 |
| **Nausea and vomiting**  n (%) | No/low burden | 991 (69.6) | 312 (73.9) | 278 (69.8) | 99 (78.0) | 50 (58.1) | 37 (48.1) |
|  | Moderate burden | - | – | – | – | – | – |
|  | Important burden | 421 (29.6) | 104 (24.6) | 120 (30.2) | 26 (20.5) | 36 (41.9) | 40 (51.9) |
|  | Missing | 11 (0.8) | 6 (1.4) | 0 (0.0) | 2 (1.6) | 0 (0.0) | 0 (0.0) |
| M±SD | | 12.0±23.0 | 9.1±19.7 | 11.9±22.6 | 8.3±20.5 | 14.0±20.6 | 22.7±29.1 |
| **Pain**  n (%) | No/low burden | 595 (41.8) | 174 (41.2) | 175 (44.0) | 57 (44.9) | 34 (39.5) | 32 (41.6) |
|  | Moderate burden | 149 (10.5) | 51 (12.1) | 34 (8.5) | 17 (13.4) | 10 (11.6) | 7 (9.1) |
|  | Important burden | 672 (47.2) | 194 (46.0) | 189 (47.5) | 52 (40.9) | 41 (47.7) | 38 (49.4) |
|  | Missing | 7 (0.6) | 3 (0.7) | 0 (0.0) | 1 (0.8) | 1 (1.2) | 0 (0.0) |
| M±SD | | 31.2±34.0 | 28.3±30.8 | 32.2±35.6 | 26.1±31.3 | 31.0±32.8 | 32.9±35.8 |
| **Dyspnoea**  n (%) | No/low burden | 727 (51.1) | 216 (51.2) | 134 (33.7) | 90 (70.9) | 49 (57.0) | 52 (67.5) |
|  | Moderate burden | - | – | – | – | – | – |
|  | Important burden | 684 (48.1) | 199 (47.2) | 264 (66.3) | 35 (27.6) | 36 (41.9) | 25 (32.5) |
|  | Missing | 12 (0.8) | 7 (1.7) | 0 (0.0) | 2 (1.6) | 1 (1.2) | 0 (0.0) |
| M±SD | | 29.3±35.2 | 27.6±33.6 | 43.2±37.6 | 14.9±26.9 | 23.1±31.3 | 19.5±31.7 |
| **Insomnia**  n (%) | No/low burden | 646 (45.4) | 199 (47.2) | 166 (41.7) | 68 (53.5) | 36 (41.9) | 32 (41.6) |
|  | Moderate burden | 299 (21.0) | 96 (22.7) | 79 (19.8) | 28 (22.0) | 18 (20.9) | 15 (19.5) |
|  | Important burden | 469 (33.0) | 123 (29.1) | 153 (38.4) | 29 (22.8) | 31 (36.0) | 30 (39.0) |
|  | Missing | 9 (0.6) | 4 (0.9) | 0 (0.0) | 2 (1.6) | 1 (1.2) | 0 (0.0) |
| M±SD | | 34.1±37.1 | 31.5±35.8 | 38.1±38.2 | 25.6±32.8 | 37.3±38.3 | 38.1±38.1 |
| **Appetite loss**  n (%) | No/low burden | 764 (53.7) | 226 (53.6) | 221 (55.5) | 73 (57.5) | 41 (47.7) | 32 (41.6) |
|  | Moderate burden | 220 (15.5) | 72 (17.1) | 57 (14.3) | 16 (12.6) | 13 (15.1) | 15 (19.5) |
|  | Important burden | 401 (28.2) | 98 (23.2) | 117 (29.4) | 36 (28.3) | 29 (33.7) | 30 (39.0) |
|  | Missing | 38 (2.7) | 26 (6.2) | 3 (0.8) | 2 (1.6) | 3 (3.5) | 0 (0.0) |
| M±SD | | 29.3±37.2 | 26.6±35.7 | 28.9±37.1 | 28.8±38.6 | 34.1±38.9 | 36.8±36.5 |
| **Constipation**  n (%) | No/low burden | 1001 (70.3) | 301 (71.3) | 292 (73.4) | 101 (79.5) | 62 (72.1) | 50 (64.9) |
|  | Moderate burden | 162 (11.4) | 53 (12.6) | 40 (10.1) | 5 (3.9) | 9 (10.5) | 12 (15.6) |
|  | Important burden | 244 (17.1) | 60 (14.2) | 66 (16.6) | 16 (12.6) | 15 (17.4) | 15 (19.5) |
|  | Missing | 16 (1.1) | 8 (1.9) | 0 (0.0) | 5 (3.9) | 0 (0.0) | 0 (0.0) |
| M±SD | | 17.8±31.4 | 15.8±29.1 | 16.8±31.2 | 12.0±28.4 | 18.2±33.0 | 20.8±32.5 |
| **Diarrhea**  n (%) | No/low burden | 1134 (79.7) | 348 (82.5) | 346 (86.9) | 76 (59.8) | 68 (79.1) | 45 (58.4) |
|  | Moderate burden | - | – | – | – | – | – |
|  | Important burden | 270 (19.0) | 66 (15.6) | 51 (12.8) | 46 (36.2) | 18 (20.9) | 31 (40.3) |
|  | Missing | 19 (1.3) | 8 (1.9) | 1 (0.3) | 5 (3.9) | 0 (0.0) | 1 (1.3) |
| M±SD | | 10.7±24.8 | 8.1±20.6 | 6.5±19.6 | 21.9±32.0 | 13.2±29.0 | 24.6±34.2 |
| **Financial difficulties**  n (%) | No/low burden | 966 (67.9) | 264 (62.6) | 280 (70.4) | 91 (71.7) | 55 (64.0) | 38 (49.4) |
|  | Moderate burden | - | – | – | – | – | – |
|  | Important burden | 417 (29.3) | 142 (33.6) | 114 (28.6) | 31 (24.4) | 28 (32.6) | 37 (48.1) |
|  | Missing | 40 (2.8) | 16 (3.8) | 4 (1.0) | 5 (3.9) | 3 (3.5) | 2 (2.6) |
| M±SD | | 16.6±29.0 | 18.5±29.0 | 16.8±30.0 | 13.9±27.7 | 17.3±27.2 | 29.8±36.6 |

- Threshold too low to calculate third category
